# Short Day–Mediated Cessation of Growth Requires the Downregulation of AINTEGUMENTALIKE1 Transcription Factor in Hybrid Aspen

**DOI:** 10.1371/journal.pgen.1002361

**Published:** 2011-11-03

**Authors:** Anna Karlberg, Laszlo Bako, Rishikesh P. Bhalerao

**Affiliations:** 1 Umeå Plant Science Centre, Department of Forest Genetics and Plant Physiology, Swedish University of Agricultural Sciences, Umeå, Sweden; 2 Umeå Plant Science Centre, Department of Plant Physiology, Umeå University, Umeå, Sweden; North Carolina State University, United States of America

## Abstract

Day length is a key environmental cue regulating the timing of major developmental transitions in plants. For example, in perennial plants such as the long-lived trees of the boreal forest, exposure to short days (SD) leads to the termination of meristem activity and bud set (referred to as growth cessation). The mechanism underlying SD–mediated induction of growth cessation is poorly understood. Here we show that the AIL1-AIL4 (AINTEGUMENTALIKE) transcription factors of the AP2 family are the downstream targets of the SD signal in the regulation of growth cessation response in hybrid aspen trees. AIL1 is expressed in the shoot apical meristem and leaf primordia, and exposure to SD signal downregulates *AIL1* expression. Downregulation of *AIL* gene expression by SDs is altered in transgenic hybrid aspen plants that are defective in SD perception and/or response, e.g. PHYA or FT overexpressors. Importantly, SD–mediated regulation of growth cessation response is also affected by overexpression or downregulation of *AIL* gene expression. AIL1 protein can interact with the promoter of the key cell cycle genes, e.g. CYCD3.2, and downregulation of the expression of D-type cyclins after SD treatment is prevented by AIL1 overexpression. These data reveal that execution of SD–mediated growth cessation response requires the downregulation of *AIL* gene expression. Thus, while early acting components like PHYA and the CO/FT regulon are conserved in day-length regulation of flowering time and growth cessation between annual and perennial plants, signaling pathways downstream of SD perception diverge, with AIL transcription factors being novel targets of the CO/FT regulon connecting the perception of SD signal to the regulation of meristem activity.

## Author Summary

Day length is a critical and robust environmental cue utilised by plants to modulate their patterns of growth to adapt to changing seasonal conditions. In perennial plants such as long-lived trees of the boreal forest, reduction in day length (short day signal/SD) induces the cessation of growth prior to the advent of winter. This developmental switch is of major adaptive significance as inability to undergo growth cessation leads to mortality in these plants. Our knowledge of how SD signal induces growth cessation is rudimentary. Here we show that *AIL1* (*AINTEGUMENTALIKE 1*), a plant-specific transcription factor, is a downstream target of the SD signal and can regulate the expression of key cell proliferation related genes. Intriguingly, the early acting components in day length–regulated processes such as flowering and growth cessation are conserved between annual and perennial plants. However our results show that the pathways downstream of short day perception diverge between these day length–controlled developmental transitions. These results have important implications for the evolution of the perennial life cycle and demonstrate how the same signal, namely day length, can regulate diverse developmental switches in annual and perennial plants.

## Introduction

The ability to adapt to changes in the environment is crucial to the survival of both animals and plants. Plants, unlike animals, are sessile organisms and have therefore evolved highly sophisticated mechanisms to anticipate seasonal changes and modulate their patterns of growth and development. Day length is one of the key environmental cues utilised by plants to anticipate seasonal changes and regulates several key developmental transitions associated with plant adaptation and reproduction. One of the most fascinating examples of this is provided by perennial plants, e.g. the long-lived trees of the boreal forest, in which the day length signal regulates the developmental transition from active growth to a more resilient dormant state prior to the onset of winter [1]. These perennial plants anticipate the approach of winter by detecting the reduction in day length (i.e. the short day signal, or SD signal) in the autumn and when the day length falls below the critical day length required for the promotion of growth, cell division in the meristems ceases [2]. The most visible indicator of short day–induced growth cessation is the formation of a bud that encloses the apical meristem and leaf primordia [3]. The importance of day length sensing for the survival of perennial plants is illustrated by the increased mortality due to delayed growth cessation in transgenic hybrid aspen plants that are unable to sense reductions in day length [4].

Intriguingly, there are numerous similarities at the regulatory level between day length mediated control of growth cessation in perennial plants and one of the most well studied developmental transitions in plants - the transition from vegetative growth to floral development. For example, key flowering time regulators such as the CONSTANS (CO), FLOWERING LOCUS T (FT) and the group of photoreceptors known as PHYTOCHROMES (PHYs) that are involved in day length mediated regulation of flowering time regulation in *Arabidopsis* [5], [6], [7], [8], are all also involved in SD–induced growth cessation in trees [4], [9], [10], [11]. In poplar species two closely related orthologs of FT (FT1 and FT2) have been found and recent analysis in hybrid aspen clone 353 indicates that FT2 could be primarily involved in SD–mediated growth cessation whereas FT1 is primarily involved in flowering [11]. In hybrid aspen (clone T89, used in this study), it has been shown that short day mediated downregulation of *FT* gene expression (*FT1* and *FT2*) triggers the induction of growth cessation whereas overexpression of *FT1* eliminates the plants' ability to respond to the SD signal and thus prevents timely growth cessation [9]. Both CO and PHYTOCHROME A (PHYA) act upstream of the *FT* genes in SD-mediated induction of growth cessation in much the same manner as in the flowering transition in *Arabidopsis* [9]. Subjecting aspen trees to conditions in which the peak of *CO* expression occurs in the dark (e.g. under SD conditions) brings about rapid downregulation of *FT2* expression leading to the induction of growth cessation response [9]. These findings indicate evolutionary conservation of the day length response pathway between annual plants such as *Arabidopsis* and perennial trees such as hybrid aspen.

Despite the evolutionary conservation of early acting components involved in day length regulated growth cessation and flowering time, considerable lacunae remain in our understanding of SD mediated regulation of growth cessation at the molecular level. Particularly, the factors targeted by SD signal downstream of the early acting components such as PHYA and the CO/FT regulon in regulating growth cessation responses remain unknown. The critical role of these hitherto unknown downstream targets of SD signal has become evident from the analysis of growth cessation response in hybrid poplar where they have been shown to be important for regulating the variation in timing of growth cessation responses [12]. Thus a key question that remains unanswered is; How does SD mediated downregulation of *FT2* expression lead to the induction of growth cessation response? Answering this question would require the identification of targets of SD signal downstream of the CO/FT regulon in trees and elucidating their role in the regulation of growth cessation responses. The network of genes involved in day length regulation of the floral transition is well defined, and downstream targets of the CO/FT pathway like *SUPPRESSOR OF OVEREXPRESSION OF CONSTANS* (*SOC1*) and floral meristem identity genes *FRUITFUL* (*FUL*) and *APETALA1* (*AP1*) are known (reviewed in [13]). In contrast the targets of the SD signal downstream of the CO/FT module in the growth cessation response can not simply be deduced from extrapolation of knowledge of floral transition related genes given the difference between the growth cessation process and floral transition.

To identify the downstream targets of the SD signal in growth cessation we have previously analysed global transcriptional changes associated with this process [14], [15]. One of the genes whose transcript is strongly downregulated during growth cessation is a *Populus* homolog of the *Arabidopsis* gene *AINTEGUMENTA* (*ANT*)[16]; the *Populus* homolog is henceforth referred to as *AINTEGUMENTALIKE1* (*AIL1*). The expression data along with a proposed role of ANT in the regulation of cell cycle [17], [18], [19] suggested that AIL1 (and most likely the other closely related members AIL2-AIL4 of this sub-family) is a potential downstream target of the SD signal transduced via the CO/FT module in the regulation of growth cessation response in perennial trees. We tested this hypothesis by investigating the regulation of *AIL1* expression by SD signal in transgenic hybrid aspen plants that are perturbed in the SD response. In a complementary approach we investigated the short day mediated regulation of growth cessation response in transgenic hybrid aspen plants that either maintain high levels of *AIL1* or *AIL3* expression even after SD treatment or have reduced expression of *AIL1*. Finally we identified downstream targets of the AIL1 transcription factor in the apex. Taken together, these analyses show that *AIL* genes are targets of the SD signal downstream of the CO/FT module and their down-regulation is necessary for short day regulated growth cessation in hybrid aspen plants.

## Results

### Downregulation of the expression of the *AINTEGUMENTALIKE (AIL)* gene family coincides with the cessation of growth and bud set following SD treatment in *Populus*

**Figure 1:**
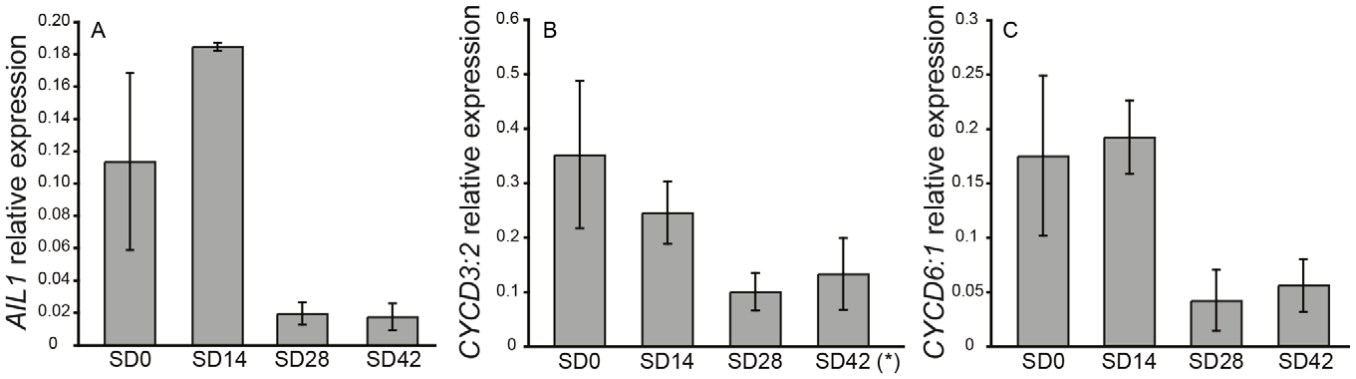
Expression of *AIL1* and D-type cyclins in the apex during short day treatment. Expression of (A) *AIL1*, (B) CYCD3:2* and (C) *CYCD6:1* in the apex of wild type hybrid aspen analyzed by real-time PCR after 0 (0SD), 14 (14SD), 28 (28SD) and 42 (42SD) days of short day treatment (8 h day). X-axis indicates time in short days and Y-axis indicates the ratio of gene expression relative to that of the reference gene (UBQ). (Error bars  =  Standard deviation of three biological replicates). *one sample excluded due to poor specificity of amplification based on melt curve analysis.

The *Populus* genome contains 13 genes belonging to the ANT-subgroup of the AP2 transcription factor family [20]. Four of these genes are here designated as *AIL1-AIL4* (*AINTEGUMENTALIKE 1-4*) as they belong to the same clade as the *Arabidopsis ANT* transcription factor (Figure S1). We investigated the expression of *AIL1* as well as the expression of the related genes *AIL2-AIL4* in the apex of hybrid aspen plants after SD treatment (Figure 1A and Figure S2). RT-PCR data indicates that *AIL1* (Figure 1A) as well as *AIL2-AIL4* expression (Figure S2) are downregulated along with that of cell cycle markers *CYCD3:2* and *CYCD6:1* after SD treatment (Figure 1B, panels B and C) and this downregulation coincides with the cessation of growth and bud set in the apex of hybrid aspen T89 trees [21].

### Downregulation of *AIL* gene expression is perturbed in hybrid aspen plants with altered SD response

**Figure 2:**
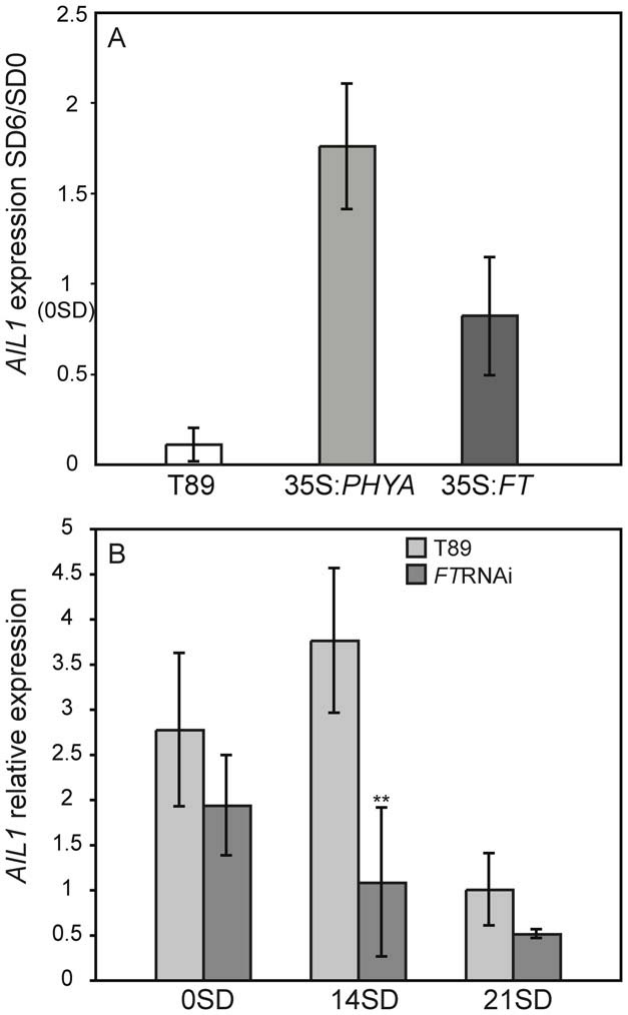
Expression of *AIL1* in the apex of transgenic lines with aberrant SD response. (A) *AIL1* expression in the apex of wild type hybrid aspen (T89), transgenic hybrid aspen overexpressing *PHYA* (35S:*PHYA*) [4] or *FT1* (35S:*FT1*) [9] after 6 weeks of SD treatment (8 h day). Expression levels (represented on Y-axis) for each genotype are normalised against their expression at SD0 (prior to the start of short day treatment). (Error bars = standard deviation of three separate experiments). (B) *AIL1* expression in the apex of wild type (T89) and *FT*RNAi [9] during the first three weeks in SD (8 h day). After two weeks (SD14) *AIL1* expression is significantly lower in *FT*RNAi compared to that in the wild type (T89) (p<0.05). X-axis indicates time in short days (SD) and Y-axis indicates the ratio of candidate gene expression relative to the average expression of two reference genes (UBQ and TIP41-like). (Error bars = Standard deviation of three biological replicates).

We then investigated the effect of perturbed SD perception or response on the regulation of *AIL* gene expression after SD treatment. For this we used transgenic hybrid aspen that are unable to respond to the SD signal due to overexpression of *PHYA* or *FT1* cDNA as well as plants that are hypersensitive to the SD signal and undergo premature growth cessation due to the downregulation of *FT* expression (*FT*RNAi) [4], [9]. Since all 4 *AIL* genes are highly similar and displayed similar expression pattern after SD treatment, we chose to perform detailed analysis of *AIL1* regulation. RT-PCR analysis indicated that the downregulation of *AIL1* expression after SD treatment is severely attenuated in the apex of *PHYA* and *FT1* overexpressors in contrast with the wild type (Figure 2A). In contrast the *FT*RNAi plants that respond more rapidly to SD treatment than wild type [9] display a stronger and earlier reduction in the expression of *AIL1* (Figure 2B). These results strongly suggest that *AIL1* expression is a potential downstream target of the SD signal transduced via the CO/FT module in cessation of growth and bud set in the apex of hybrid aspen.

### *AIL1* expression in hybrid aspen apex is confined to zones of actively dividing cells

**Figure 3:**
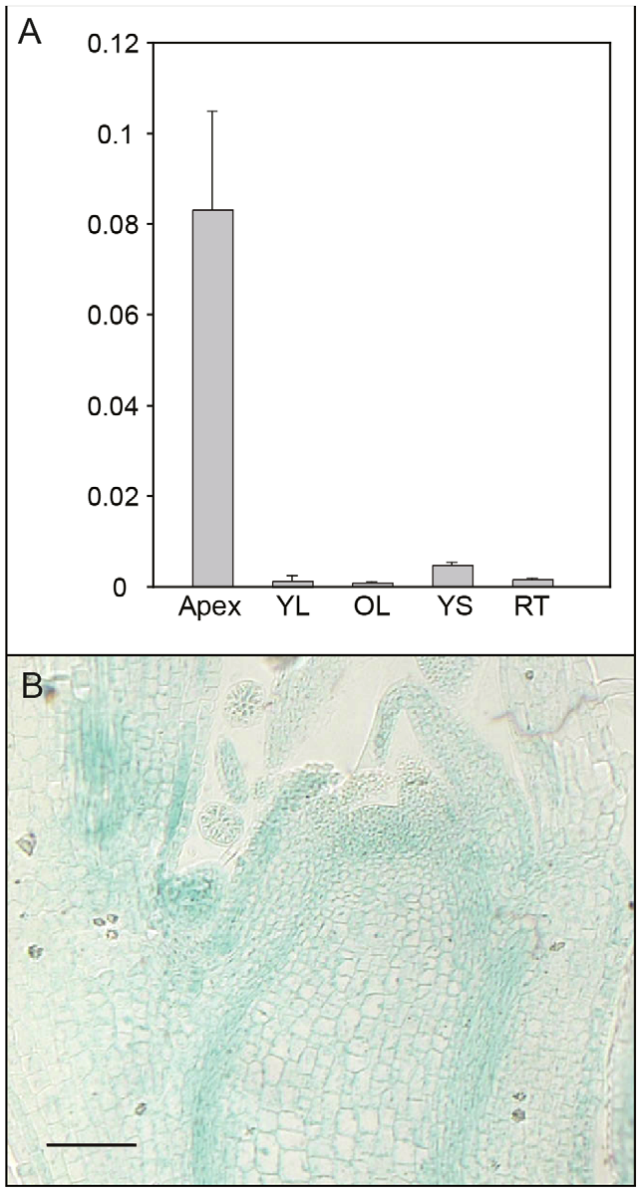
Analysis of tissue-specific *AIL1* expression pattern. (A) *AIL1* expression across the different tissues; apex, young leaf (YL), old leaf (OL), young stem (YS), old stem (OS) and root tip (RT) in tissue culture grown wild type hybrid aspen. All expression values (on Y-axis) are ratios of *AIL1* expression relative to the reference (UBQ). (Error bars = Standard deviation of three biological replicates) (B) GUS activity in actively growing hybrid aspen apex expressing the *uid*A (GUS) gene under control of the *AIL1* promoter.

We further investigated expression of *AIL1* in different tissues and found that *AIL1* is primarily expressed in the apical region of hybrid aspen (Figure 3A). Since the downregulation of *AIL1* gene expression was closely associated with cessation of growth and bud set, we analysed the domain of *AIL1* gene expression in the apex. For this analysis we generated transgenic hybrid aspen expressing a transcriptional fusion between 2.5 Kb upstream sequence of *AIL1* gene from *Populus trichocarpa* and the *uid*A (*b-glucoronidase*/*GUS*) reporter gene. In the transgenic hybrid aspen, the reporter gene expression was mostly confined to the zone of dividing cells in the apex, the provascular tissues and the leaf primordia (Figure 3B). The expression pattern of pAIL1:*UidA* reporter construct correlates well with the previously described expression pattern of *CYCA1* which serves as a marker of dividing cells in the apex of hybrid poplar plants [22] indicating that *AIL1* expression is associated primarily with cell proliferation.

### Perturbing *AIL* gene expression affects SD–mediated regulation of growth cessation response

**Figure 4:**
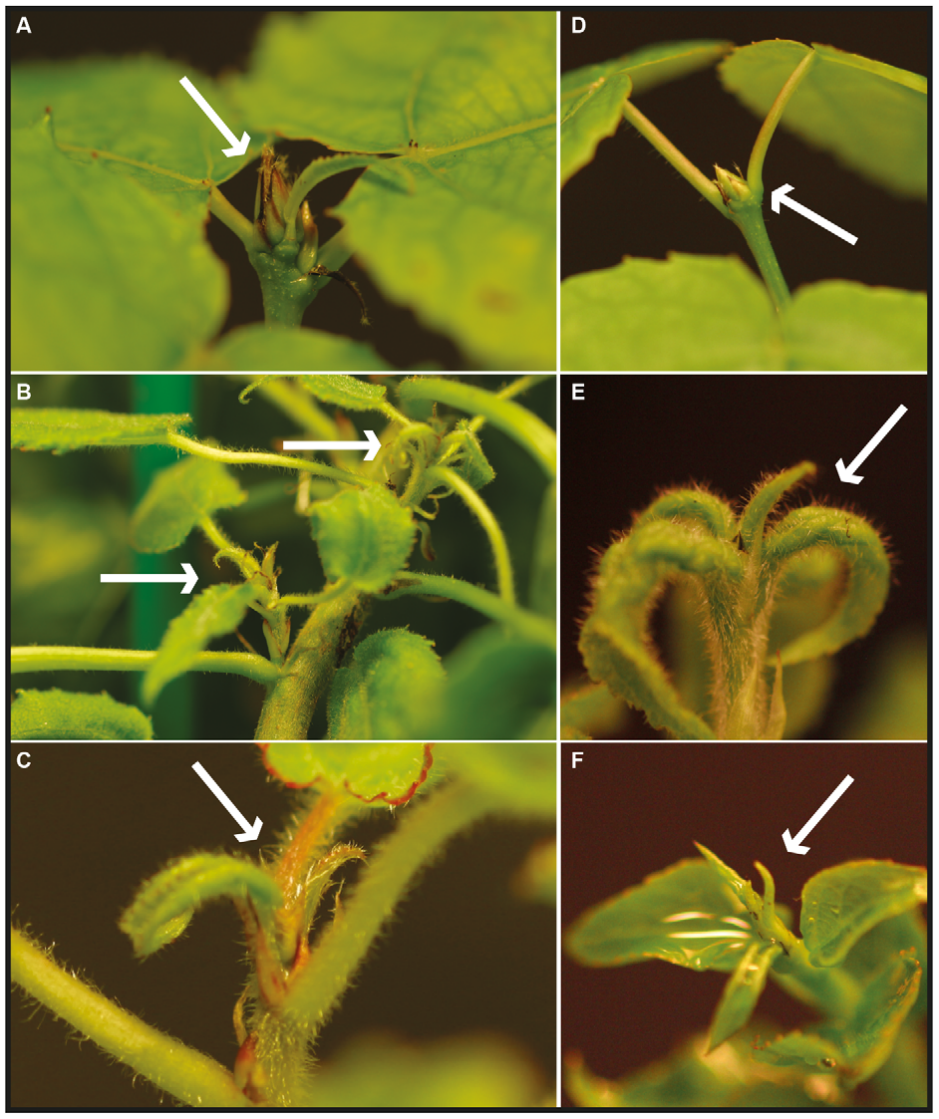
Bud formation in wild type, *AIL1* (*AIL1*oe), and *AIL3* (*AIL3*oe) overexpressing transgenic hybrid aspen plants after 6 weeks in short days. Six weeks of SD-treatment (8 h light) leads to bud formation in wild type T89 (A), whereas no bud set is observed in *AIL1*oe line 2B or 3B (B and C). Similarly in SD treatment consisting of 14 hours day length, bud formation is observed after 6 weeks SD-treatment in apices of wild type T89 (D) whereas no bud set is observed in *AIL3*oe line 9 (E) or *AIL3*oe line 10 (F). Position of the apex is indicated by white arrows.

Analysis of *AIL1* gene expression suggested that its downregulation could be important for the SD mediated cessation of growth and bud set. We tested this hypothesis by generating transgenic hybrid aspen that would maintain high levels of *AIL1* expression even after SD treatment in contrast with the wild type by expressing *AIL1* cDNA under the control of the 35S promoter (these transgenic lines are henceforth referred to as *AIL1*oe lines). Several independent lines were obtained and tested for high expression of *AIL1* (Figure S3A shows data for two chosen lines); two lines were chosen for detailed analysis of their response to SD treatment. Unlike the wild type plants that undergo growth cessation and form an apical bud after 6 week of SD treatment, the apices of *AIL1*oe fail to undergo proper growth cessation and bud set after 6 weeks of SD treatment (Figure 4A–4C). We also generated transgenic hybrid aspen overexpressing *AIL3* (Figure S3) to investigate whether other members of the gene family share the same function (these transgenic lines are henceforth referred to as *AIL3*oe lines). *AIL3*oe display a similar phenotype to *AIL1*oe during SD treatment (Figure 4D–4F).

We also investigated the effect of downregulation of *AIL* gene expression on SD mediated bud set. The functional redundancy between *AIL* genes suggested by similar regulation and effect on bud set in *AIL1*oe and *AIL3*oe plants lead us to generate transgenic hybrid aspen plants in which the expression of all 4 *AIL* genes was targeted for downregulation using artificial microRNA (amiRNA). Two amiRNA constructs (255 and 256) were expressed in hybrid aspen and two lines (255-6 and 256-23) with reduced expression of *AIL1* gene expression (Figure S4) were selected for further analysis of growth cessation response. The transition from active growth to bud set after SD treatment in the wild type and lines 255-6 and 256-23 was investigated using a method separating different stages of bud development [23]. Compared to the wild type, the lines 255-6 and 256-23 displayed a more rapid transition from active growth to bud set and a majority of the plants in the two transgenic lines made the transition to intermediate and late stage of bud set at least a week (or more) earlier than the wild type plants after SD treatment (Figure S5). This data along with the perturbed growth cessation response in *AIL1*oe and *AIL3*oe plants indicate that downregulation of *AIL* gene expression is necessary for SD mediated growth cessation response.

### AIL1 is the downstream target of SD signal in the activation of growth cessation responses

**Figure 5:**
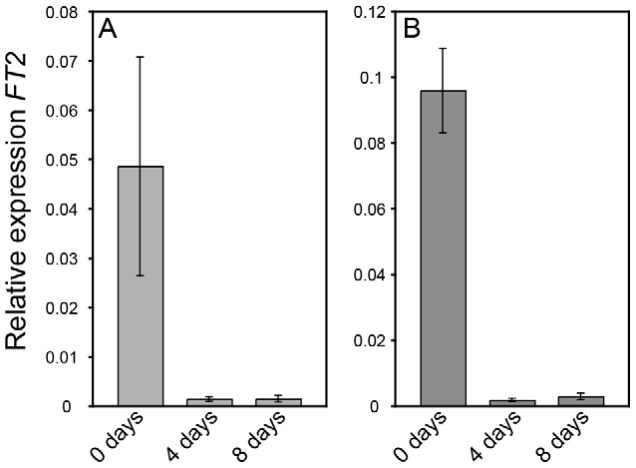
*FT2* expression is rapidly downregulated in wild type (T89) and *AIL1*oe after transfer to short days. RT-PCR analysis of *FT2* transcript level in the leaves of the wild type (A) and *AIL1oe* (B) after 0, 4 and 8 short days (8h day). Y-axis indicates the ratio of *FT2* expression relative to that of the reference gene (UBQ) and time in short days is shown on the X-axis. (Error bars: Standard deviation of three biological replicates.)

The altered growth cessation responses in *AIL1*oe plants could either be due to the failure of these plants to perceive the short day signal or to a failure in properly responding to it. To distinguish between these two possibilities we compared the response of *FT2* expression to SD treatment in the leaves of wild type and *AIL1*oe. Downregulation of *FT2* expression in the leaves is the earliest known marker for the detection of the SD signal and recent results have implicated its downregulation in SD mediated growth cessation [9], [11], [12]. Our data show that both the wild type (Figure 5A) and *AIL1*oe lines (Figure 5B) exhibit similar decreases in their levels of *FT2* transcripts following SD treatment. This result indicates that unlike *FT* genes, *AIL1* does not act early in SD response but is rather a downstream target of the SD signal.

### AIL1 is a potential regulator of D-type cyclin gene expression

**Figure 6:**
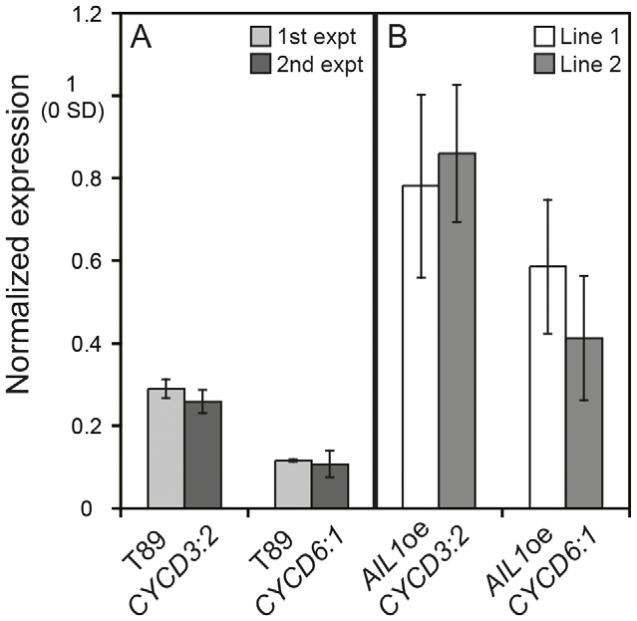
Differential regulation of D-type cyclin expression in the *AIL1*oe lines compared to the wild type (T89) after SD-treatment. (A) Expression of *CYCD3:2* and *CYCD6:1* in T89 after 6 weeks SD (8 h day). Results from two separate experiments are shown. All expression values normalised to expression at 0 SD. (Error bars: SD of three technical replicates). (B) Expression of *CYCD3:2* and *CYCD6:1* in *AIL1oe* after 6 weeks SD. Results from two lines are shown. Expression values normalised to expression at 0 SD. (Error bars = Standard deviation of three biological replicates).

The expression of several cell proliferation related genes, e. g. D-type cyclins, that are key cell cycle regulators [24], [25], [26], [27] is downregulated in a similar manner to *AIL* genes during SD mediated cessation of growth in hybrid aspen [15], Figure 1B, 1C). We therefore investigated whether the AIL1 transcription factor could be involved in the regulation of the D-type cyclin genes and if so whether their expression is perturbed in *AIL1*oe lines after SD treatment. Therefore we analysed the expression of two D-type cyclins, *CYCD3:2* and *CYCD6:1* after SD treatment in *AIL1*oe plants after 6 weeks of SD treatment. Our RT-PCR data (Figure 6) showed that while the expression of *CYCD3:2* and *CYCD6:1* is downregulated in the wild type after 6 weeks of SD treatment, this was not the case in the *AIL1*oe plants. This result indicates that *AIL1* could be involved in the regulation of D-type cyclins and the failure to downregulate *AIL1* expression after SD treatment leads to a corresponding failure to downregulate the expression of these key cell cycle regulators.

### AIL1 transcription factor can interact with D-type cyclin promoters

**Figure 7:**
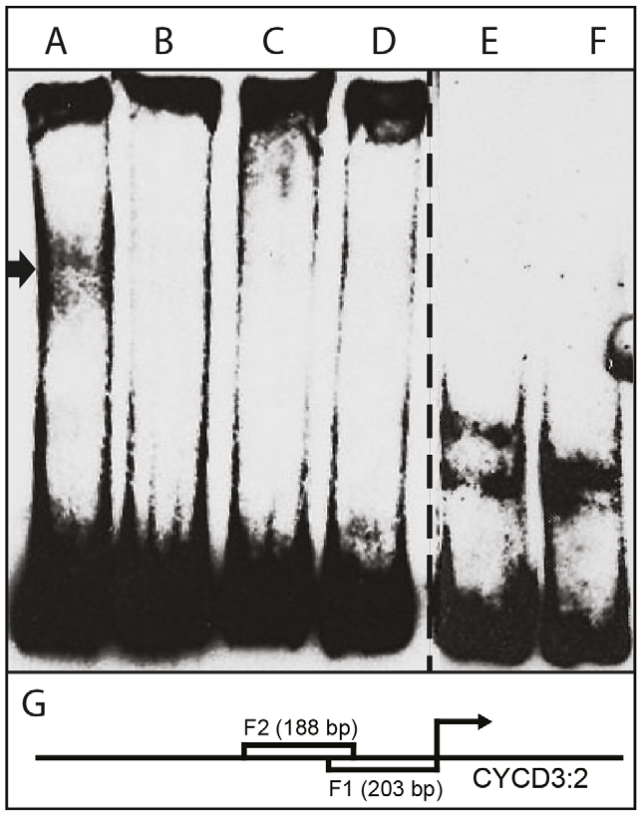
Interaction of HA-tagged AIL1 with *CYCD 3:2* promoter fragment by electrophoretic mobility shift assay (EMSA). (A) AIL1 + 5 fmol biotin- labeled CYCD 3:2 promoter fragment F1, (B) AIL1 + biotin- labeled CYCD 3:2 promoter fragment F1+ 500 fmol unlabeled F1, (C) AIL1 + 5 fmol biotin- labeled CYCD 3:2 promoter fragment F2, (D) AIL1 + Biotin- labeled CYCD 3:2 promoter fragment F2+ 500 fmol unlabeled F2, (E) biotin-labeled F1, (F) biotin-labeled F2 and (G) F1 and F2 in relation to the start codon of CYCD 3:2. Lanes A-D have additional non-specific competitor added to the reaction mix. Cell extracts from protoplast expressing HA-tagged AIL1 were used for gel-shift analysis. Additional lanes between D and F on the gel are omitted from the picture.

The observation that *CYCD3:2* and *CYCD6:1* expression after SD treatment is perturbed in *AIL1*oe plants prompted us to investigate whether the AIL1 transcription factor can interact with *CYCD* promoters from hybrid aspen using electrophoretic mobility shift assays (EMSA). We expressed HA-tagged AIL1 protein in *Arabidopsis* protoplasts and used the extracts in gel shift assays using 3 different different fragments from a hybrid aspen *CYCD3:2* promoter (results from two fragments are shown here). Our data show that extracts containing AIL1 protein specifically display a gel shift with the promoter fragment consisting of 200 bp of sequence situated upstream of the start codon of *CYCD3:2* (Figure 7). Together with the *CYCD3:2* and *CYCD6:1* gene expression data, the gel-shift analysis strongly suggests these cyclin genes might be potential downstream targets of the AIL1 transcription factor in hybrid aspen.

## Discussion

Short day mediated cessation of growth and budset prior to the onset of winter is a key developmental transition that is critical to the survival of perennial plants in boreal forest [1]. In this work, we identify *AIL* genes belonging to the AP2 transcription factor family as downstream targets of the SD signal transduced via the CO/FT module and that downregulation of their expression is necessary for cessation of growth and bud set in hybrid aspen.

In poplar, there are 4 closely related *AIL* genes and our data indicates that all four *AIL* genes could have similar function at least in SD mediated growth cessation response as suggested by the similar phenotypes of *AIL1* and *AIL3* overexpressing plants as well as in plants in which these genes are targeted for downregulation. However we cannot exclude the possibility that there could be functional differences between the different *AIL* genes with respect to other biological processes as we have not been able to specifically downregulate individual genes of the *AIL* family and study the effect on growth and development so far. It is particularly important to note in this respect that even closely related genes can diverge both in expression profiles and at a functional level as suggested by the careful analysis of FT1 and FT2 in poplar species which indicate that FT1 could be primarily involved in reproductive growth whereas FT2 controls growth cessation [11].

Several observations suggest that the downregulation of *AIL* gene expression following SD treatment is necessary for the activation of growth cessation responses. *AIL1* (and most likely other genes of this family as well) is primarily expressed in dividing and meristematic cells in hybrid aspen (Figure 3) and the downregulation of their expression coincides temporally with the SD-mediated induction of growth cessation responses in hybrid aspen (Figure 1 and Figure 4), including the termination of elongation growth, bud set and the downregulation of core cell cycle genes such as the D-type cyclins (Figure 1). Furthermore, *AIL1* downregulation after SD treatment is attenuated in *FT* or *PHYA* overexpressors (Figure 2) that fail to respond properly to SD treatment [4], [9]. Importantly, growth cessation response is perturbed when *AIL1* or *AIL3* expression is maintained at high levels even after SD treatment and earlier bud set is observed in transgenic hybrid aspen with reduced expression of *AIL1* (Figure 4 and Figure S5). All of these results are consistent with the *AIL* genes being downstream targets of the SD signal in the control of the growth cessation response.

Three hypotheses can be proposed to explain the role of *AIL* genes in SD mediated control of growth cessation response and why growth cessation response is perturbed when the *AIL1 or AIL3* expression is maintained at high level even after SD treatment as in AIL1oe and AIL3oe lines. Firstly, the *AIL* genes could act upstream of *FT2*, in which case the increased expression of *AIL1* as in *AIL1oe* could counteract the downregulation of *FT2* by the SD signal. However, this hypothesis is incompatible with the observation that the downregulation of *FT2* subsequent to SD treatment proceeds as normal in *AIL1*oe lines (Figure 5). Alternatively, the *AIL* genes could act independently of *FT2* and their increased expression in *AIL1*oe and *AIL3*oe could prevent the downregulation of the targets of *FT2* following SD treatment. Alternatively, the *AIL* genes are the targets of SD signal downstream of CO/FT regulon leading to their downregulation after SD treatment. Our results support the latter hypothesis because SD treatment results in the downregulation of the *AIL* gene expression and this downregulation of *AIL* gene expression is severely attenuated in plants that overexpress *FT1*. Further evidence for the connection between the CO/FT regulon and *AIL1* expression was obtained by analysis of *FT*RNAi lines that respond more rapidly than the wild type to SDs and in these lines, *AIL1* expression is significantly reduced compared to wild type after 2 weeks of SD treatment (Figure 2B). Finally the downregulation of the *AIL1* expression leads to earlier transition from active growth to bud set strongly suggesting that the *AIL* genes are the downstream targets of the SD signal. Thus our results suggest a mechanism in which *AIL* genes act downstream of the CO/FT regulon and that downregulation of *AIL* gene expression culminates in growth cessation and bud set after SD treatment.

FT has been shown to act as a transcriptional co-regulator in *Arabidopsis* [28]. In poplar species, two *FT* genes are present of which *FT2* is rapidly downregulated after SD treatment; thus FT2 could either directly regulate *AIL* at the transcriptional level in hybrid aspen or alternatively downstream targets of FT2 could regulate *AIL* gene expression. Our data supports the latter suggestion because the kinetics of downregulation of *FT2* and *AIL* gene expression subsequent to SD treatment is not consistent with direct regulation of *AIL* gene expression by FT2. While *FT2* is typically downregulated within 3–7 days in the leaves after the commencement of SD treatment ([9], [12], Figure 5), it takes 2–3 weeks until downregulation of the *AIL* genes becomes apparent in the apex (Figure 1). Moreover, induction of *FT2* in the leaves has little effect on expression of most of *AIL* genes [11], which might again suggest an indirect regulation of *AIL* expression by FT2. Thus these results suggest that there may be one or more genes that are direct targets of FT2 and act upstream of the *AIL* genes regulating their expression in the apex. Determining the identity of these targets of FT2 and regulators of *AIL* expression in the apex is an important objective for future research in this area. The downstream targets of FT in daylength mediated regulation of flowering time such as *SOC1* and the floral meristem identity genes *FUL* and *AP1* [13] are well known. However these are unlikely to be the targets of the CO/FT regulon in the regulation of the *AIL* genes in the apex unless the tree homologs of these genes have acquired novel functions and have been recruited to regulate meristem activity by controlling *AIL* gene expression.

*AIL1* is expressed in dividing cells (Figure 2), can potentially interact with the promoters of D-type cyclins (Figure 7) and maintaining high level of *AIL* expression prevents the downregulation of D-type cyclin expression after SD treatment (Figure 6) suggesting that AIL1 has a role in regulation of key cell cycle regulators. Indeed, data from *Arabidopsis* also shows that the putative AIL1 ortholog ANT can positively regulate cell division as its overexpression leads to increased duration of cell division [17]. We therefore propose that the downregulation of *AIL* gene expression after SD treatment leads to the downregulation of a subset of D-type cyclins such as *CYCD3:2* and *CYCD6:1*. The downregulation of the expression of core cell cycle regulators such as the abovementioned cyclins would then culminate in cessation of growth and bud set. However it is unlikely that the D-type cyclins are the only targets of *AIL1* because the expression of several other cell cycle genes is also downregulated after SD treatment [15]. Additionally transcriptional network analysis indicates that several other cell cycle genes might be regulated by the *AIL1* transcription factor [29]. Moreover, preliminary investigations suggest that altering *CYCD3:2* expression alone is not sufficient to activate the growth cessation response.

Substantial progress has recently been made in understanding how SD signal is perceived, and downregulation of *FT2* expression after SD treatment has been identified as a key early event in the induction of growth cessation response [9], [11]. However, the components targeted by SD signal downstream of the CO/FT regulon in the induction of growth cessation response have remained elusive, especially factors that would link the downregulation of *FT2* expression to cessation of growth. Indeed analyses of hybrid poplar clones that differ in timing of bud set have suggested an important role for such factors in differential growth cessation [12]. Our finding that the *AIL* genes are the targets of the SD signal that is transduced via the CO/FT module in growth cessation response and bud set therefore represents an important step in elucidating the mechanism underlying this key developmental transition in perennial plants as this links the CO/FT module to the regulation of cell cycle through the *AIL* genes in SD mediated cessation of growth and bud set.

The CO/FT module is an important component of the molecular machinery that allows plants to respond to changes in day length, and its role in day length mediated control of flowering time is well established [30]. Therefore it was not surprising that the same CO/FT module is also involved in controlling the timing of SD-mediated growth cessation in perennial trees, as this is another key developmental transition that is regulated by the day length signal. However, given that flowering and growth cessation processes are distinct morphologically, it appears unlikely that the downstream targets of this module in the regulation of flowering would be the same as those involved in the growth cessation response. Our findings suggest that the *AIL* transcription factors, which have the potential to regulate the expression of cell cycle genes, were co-opted at some point in evolutionary history to serve as mediators of the day length signal. This co-option would have allowed the versatile CO/FT module to regulate a novel developmental transition. These results demonstrate that an evolutionary “mix and match” strategy involving combining different regulatory modules can allow a small number of regulatory modules to control a wide range of diverse biological processes. In conclusion, our data demonstrates the divergence of the regulatory pathway downstream of the conserved CO/FT module between day length controlled floral transition and growth cessation response and identifies *AIL1* as a potential regulator of cell cycle related genes and a novel target of the short day signal downstream of the CO/FT module in regulation of growth cessation in perennial trees.

## Materials and Methods

### Plant material and growth conditions

Cuttings of hybrid aspen (*Populus tremula x tremuloides*) clone T89 (wild type) and the transgenic lines were grown in half-strength Murashige/Skoog medium (½ MS) under sterile conditions for approximately 4 weeks and then transferred to soil. After four weeks in greenhouse the plants were moved to growth chambers (18 hour light/6 hour night, 20°C). After one week the chamber settings were shifted to short day conditions (8 hour light/16 hour night, 20°C or 14 hour light/10 hour night, 20°C). Growth cessation was determined by measurement of elongation growth and/or bud set. Pictures of apices to assess bud formation were taken using Canon EOS digital camera. For tissue specific expression analysis of AIL genes, samples were taken from tissue culture grown plants 4 weeks after cuttings were transferred to new media.

### Identification of AIL-genes and phylogenetic analysis

AIL-genes were identified by blasting the *Arabidopsis* AINTEGUMENTA gene (AT4G37750) against the *Populus* genome. Gene models (http://genome.jgi-psf.org/Poptr1_1/Poptr1_1.home.html) were manually chosen based on intron-exon structure (JGI protein ID for each model can be found in Figure S1). Sequences were aligned and a bootstrapped phylogenetic tree generated using ClustalX [31]. The phylogenetic tree was visualised using TreeView (http://darwin.zoology.gla.ac.uk/~rpage/treeviewx/).

### Generation of *AIL1*oe and *AIL3*oe lines

The full length cDNA for *AIL1* transcription factor was cloned into the donor vector pDONR201 (Invitrogen.com) before transfer into the destination vector pK2GW7 [32]. The resulting vectors were introduced into agrobacterium GV3101pmp90RK [33] followed by the transformation of hybrid aspen clone T89 [34]. The same strategy was used to generate *AIL3*oe lines with the exception of entry clone construction that in this case was performed using the pENTR/D-TOPO cloning kit (Invitrogen.com).

### GUS promoter analysis

The *AIL1* promoter was amplified using the primers: FW: CACCCGGGGAATGATAGGCTGACAA and RP:CCCAAAATCTTGCCTACTTCC and cloned into the pENTR/D-TOPO vector (Invitrogen.com). The fragment was transferred into the pK2GWFS7 binary vector [32]. The construct was transformed into hybrid aspen using *Agrobacterium* mediated transformation as described before [34]. Apices from transgenic lines expressing the reporter gene were collected from greenhouse grown trees approx. 5 weeks after potting. The apices were incubated approx. 3 h at 37°C in GUS-solution (1 mm X-gluc, 1 mm K_3_Fe(CN)_6_, 1 mm K_4_Fe(CN)_6_, 50 mm sodium phosphate buffer (pH 7.0), and 0.1% (v/v) Triton X-100). The samples were then rinsed with water, dehydrated to 50% (v/v) ethanol, fixed for 10 min in FAA (5% (v/v) formaldehyde, 5% (v/v) acetic acid, and 50% (v/v) ethanol), and cleared in 100% (v/v) ethanol. Once cleared, the samples were embedded in LR-White/10% PEG 400 resin in polypropylene capsules (TAAB) The apices were then sectioned on a Microm HM350 microtome (Microm International GmbH, Germany) at approx 20 µm, floated on water, heat-fixed to glass slides, mounted in Entellan neu (Merck, Germany) Sections were visualized with Zeiss Axioplan light microscope and captured with a digital camera, AxioCam together with the Axiovision 4.5 software (Zeiss, Germany).

### RNA isolation and real-time PCR analysis

Total RNA from poplar apices was extracted using the Aurum Total RNA kit (Bio-Rad). Care was taken to collect tissue samples for RNA isolation at the same time of the day (usually between 13–16 PM) for each experiment. 100–500 ng of RNA was DNase treated with RNase free DNaseI (Fermenta) and used for cDNA synthesis using iScript cDNA synthesis kit (BioRad) or qScript cDNA synthesis kit (Quanta BioSciences). Reference genes were validated using GeNorm Software [35]. The reference gene chosen was UBQ in all experiments except for the analysis of the overexpression of *AIL1* and *AIL3* in the AIL1oe and AIL3oe lines, where 18S rRNA was used as the reference gene. Analysis of expression in *FT*RNA*i* used two reference genes, UBQ and TIP-41 like. SYBR green (Bio-Rad or Quanta BioSciences) was used as non-specific probe in all reactions and relative expression values were calculated using the Δ-ct-method [9]. A complete list of primers used in RT-PCR analysis can be found in Table S1.

### Generation of transgenic hybrid aspen plants with reduced expression of AIL genes

To downregulate the expression of *AIL* genes, artificial microRNAs were designed using the online tools at http://wmd.weigelworld.org/cgi-bin/mirnatools. Briefly primers (Table S3) were used to generate artificial microRNAs directed against all the 4 *AIL* genes and cloned into the plant transformation vector pK2GW7 according to the cloning protocol at http://wmd3.weigelworld.org/. Two different miRNA contructs (named 255 and 256) were made and transformed into hybrid aspen clone T89 as described earlier. Following transformation several hybrid aspen lines with reduced expression of *AIL1* were obtained and one line each for the two constructs 255 and 256 were selected for the analysis of bud set after SD treatment (lines 255-6 and 256-23).

### Analysis of bud set in hybrid aspen plants with reduced expression of AIL genes

Bud set was scored using the method described by [23]. We used a score of 3 to indicate active growth (complete lack of bud set) and 0 to indicate a completely closed bud and score of 2 or 1 to indicate intermediate stages. For this analysis, bud set was scored every 7 days in a minimum of 5 or more plants for a period of 7 weeks.

### Generation of HA-tagged AIL1 and overexpression in Arabidopsis protoplasts

AIL1 full length cDNA was amplified using the following primers: pttAIL1(EcoRI) FW- CATGGAATTCATGAAATCTACGGGTGATAA and pttAIL1(SalI) RP-CATGGTCGACTTCTCCTTTTCCTTGGTTCATGC. The resulting fragments were cloned into pRT104-3xHA [36]. Transfection into *Arabidopsis* protoplasts were performed as described [36], [37] using 8 µg of purified plasmid. Cells were lysed in a lysis buffer containing 25 mM Tris-HCL (pH 7.5), 50 mM KCl, 1 mM EDTA, 10% Glycerol 1 mM DTT, 0.1% Igepal and 1X PIC (Protease Inhibitor Cocktail). After centrifugation the supernatant was collected and immediately frozen in liquid nitrogen. The expression of the HA-tagged AIL1 protein was confirmed with western blot and resulting cell extracts were used for subsequent analysis.

### Generation of labeled CycD3:2 promoter fragments

CycD3:2 promoter sequences were identified using the JGI populus genome database (http://genome.jgi-psf.org/Poptr1_1/Poptr1_1.home.html). Approx. 200 base pair fragments were amplified using primers specified in Table S2. The fragments were gel-purified using E.Z.N.A. Gel Purification Kit (Omega Bio-Tek) followed by phenol-chloroform extraction and ethanol precipitation prior to use in gel-shift assays. Five pmol of purified fragments were biotin labeled using the Biotin 3′ End DNA Labeling Kit (Pierce). Labeling and labeling efficiency determination was performed according to the manufacturers recommendation.

### Electrophoretic mobility shift assay

The biotin-labelled promoter fragments were mixed with protoplast cell extracts containing AIL1-HA or control extracts from non-transfected protoplasts. For the binding reaction the following conditions were used: 10 µl protoplast cell extract, 0.5 µl biotin-labelled DNA (10 fmol/µl), 0.4 µl non-specific competitor (poly (dI:dC), 1 mg/ml), 0.5 µl BSA (20 mg/ml) and lysis buffer to a total of 20 µl. For specific competition, 500 fmol non-labelled fragment was added to the reaction. Binding was performed on ice for 10 min followed by 30 min in room temperature. The samples were run on a non-denaturing polyacrylamide gel (5%-0.5xTBE) and transferred to a Hybond N+ membrane (GE Healthcare, Sweden). Crosslinking and detection was performed using the LightShift Chemiluminescent EMSA kit (Pierce.com).

## Supporting Information

Figure S1 Phylogenetic analysis of the ANT family of AP2 transcription factors in *Arabidopsis* and *Populus*. Arabidopsis *AINTEGUMENTA* (*ANT*) groups with four *Populus* genes, which were named *AINTEGUMENTALIKE 1-4* (*AIL1-AIL4*). *AIL1* and *AIL3* analysed in detail are marked in bold letters.
(TIF)

Figure S2 Expression of *AIL* genes is downregulated after short day treatment during growth cessation. Expression of *AINTEGUMENTALIKE* genes (*AIL1-AIL4*) was analysed in the apex of wild type hybrid aspen after 6 weeks of SD treatment (8h day). Y-axis indicates the expression levels after 6 weeks of short day treatment normalized to the level prior to the start of short day treatment (SD 0). In each case average from three independent experiments is shown and error bars represent standard deviation.
(TIF)

Figure S3 Expression of *AIL1* and *AIL3* in transgenic hybrid aspen. Expression in the apex of *AIL1* in the wild type hybrid aspen (T89) and two transgenic lines (*AIL1*oe line 2B and 3B) expressing *AIL1* cDNA under the control of 35S promoter. Y-axis shows the transcript levels of *AIL1* relative to that of the reference gene (18S rRNA). Data from 2 independent experiments is shown. (B) Expression of *AIL3* in wild type hybrid aspen (T89) and two transgenic lines (*AIL3*oe line 9 and line 10) expressing the *AIL3* cDNA under the control of 35S promoter. Y-axis shows the ratio of *AIL3* expression relative to that of the reference gene (18S rRNA). The expression values are average of 3 biological replicates and error bars represent the standard deviation for the three biological replicates.
(TIF)

Figure S4 Expression of *AIL1* in amiRNA expressing lines. A). Expression of *AIL1* in apices of tissue culture grown wild type (T89) and amiRNA expressing hybrid aspen (255-6). A). Expression of *AIL1* in apices of tissue culture grown wild type (T89) and amiRNA expressing hybrid aspen (256-23). Expression values are median of three technical replicates.
(TIF)

Figure S5 Analysis of bud set in amiRNA expressing lines. Analysis of bud set phenotype in wild type hybrid aspen (T89), and amiRNA lines 255-6 and 256-23. The transition from active growth to completion of bud set was divided into 4 stages where 0 is a completely developed bud and 3 correspond to an actively growing apex. Number of plants used for each genotype are: T89 n = 5, 255-6 n = 7 and 256-23 n = 6. X-axis denotes weeks in short days and Y-axis denotes number of plants at a particular stage of bud set transition. Colors specifying the stage of bud set are denoted. (Day length = 14h)
(TIF)

Table S1 Real-time PCR primer sequences. *Please note that cross-reactivity could occur between primer pairs used for the detection of *AIL1* and *AIL2* gene expression.
(DOCX)

Table S2 Primers for CYCD3:2 promoter fragments.
(DOCX)

Table S3 Primers for amiRNA construction.
(DOCX)
