# Supplementary figures and images for: Short Day–Mediated Cessation of Growth Requires the Downregulation of AINTEGUMENTALIKE1 Transcription Factor in Hybrid Aspen
Source: PLoS Genet. 2011 Nov 3;7(11):e1002361. doi: 10.1371/journal.pgen.1002361 (PMC3207903; doi:10.1371/journal.pgen.1002361)

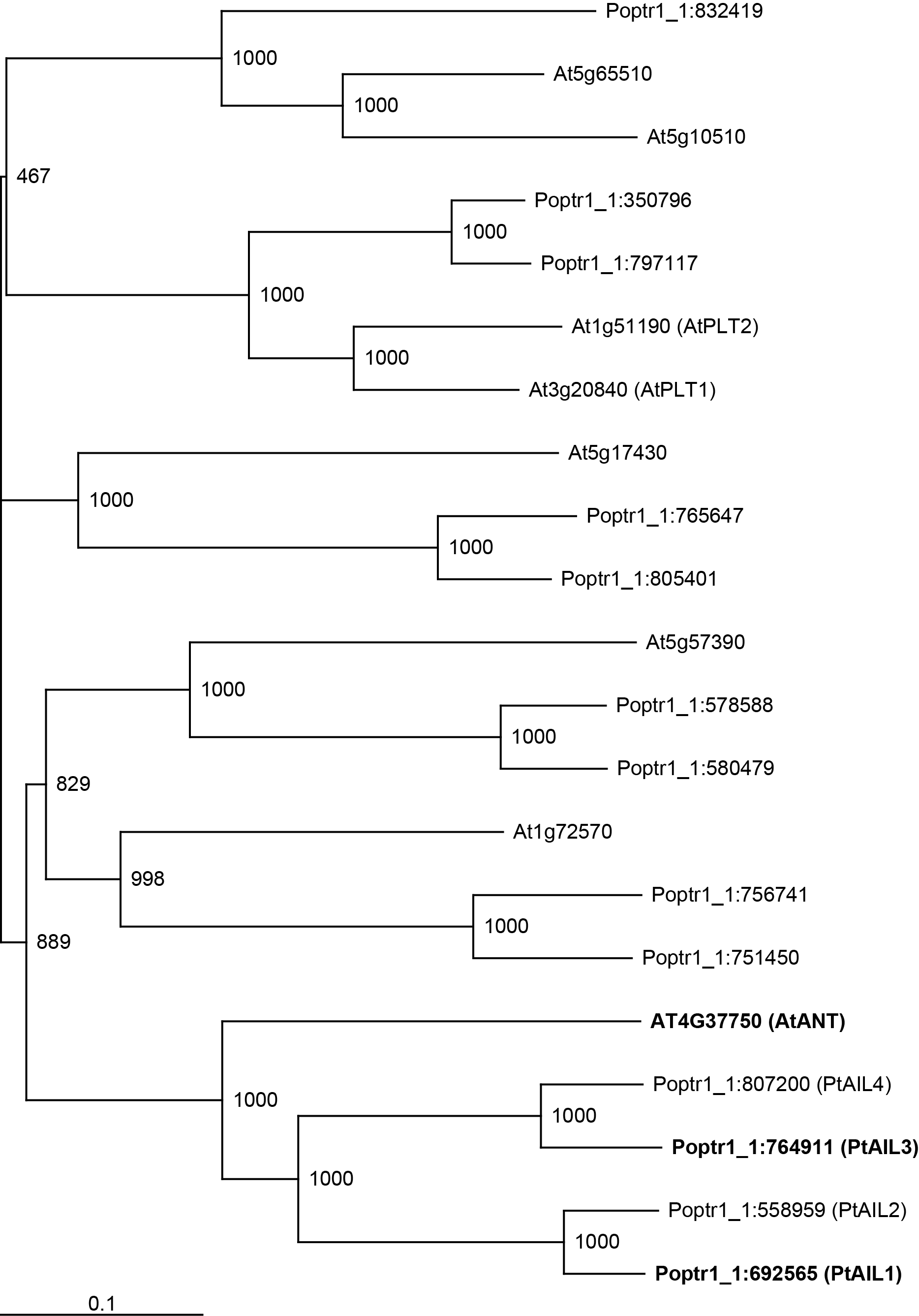

Supplement: Figure S1 — Phylogenetic analysis of the ANT family of AP2 transcription factors in Arabidopsis and Populus. Arabidopsis AINTEGUMENTA (ANT) groups with four Populus genes, which were named AINTEGUMENTALIKE 1-4 (AIL1-AIL4). AIL1 and AIL3 analysed in detail are marked in bold letters. (TIF) [file pgen.1002361.s001.tif]

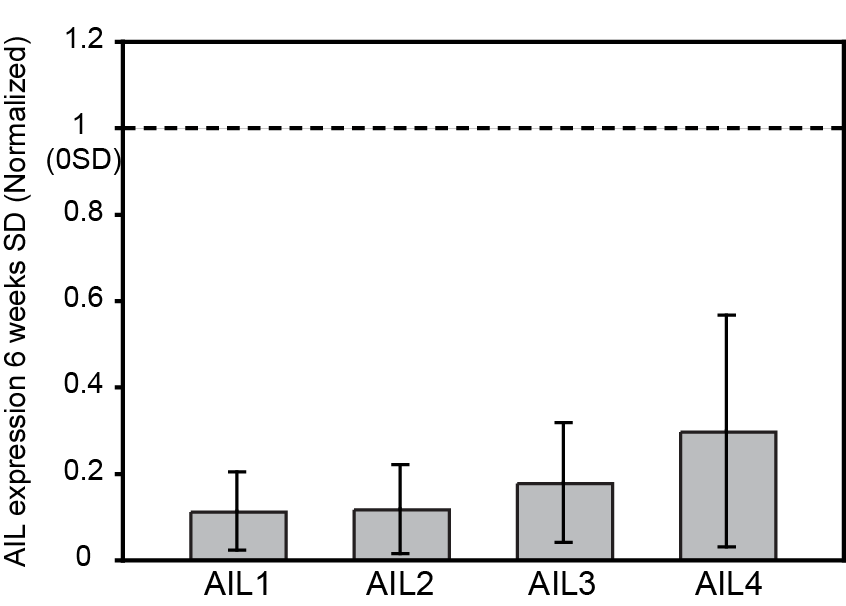

Supplement: Figure S2 — Expression of AIL genes is downregulated after short day treatment during growth cessation. Expression of AINTEGUMENTALIKE genes (AIL1-AIL4) was analysed in the apex of wild type hybrid aspen after 6 weeks of SD treatment (8h day). Y-axis indicates the expression levels after 6 weeks of short day treatment normalized to the level prior to the start of short day treatment (SD 0). In each case average from three independent experiments is shown and error bars represent standard deviation. (TIF) [file pgen.1002361.s002.tif]

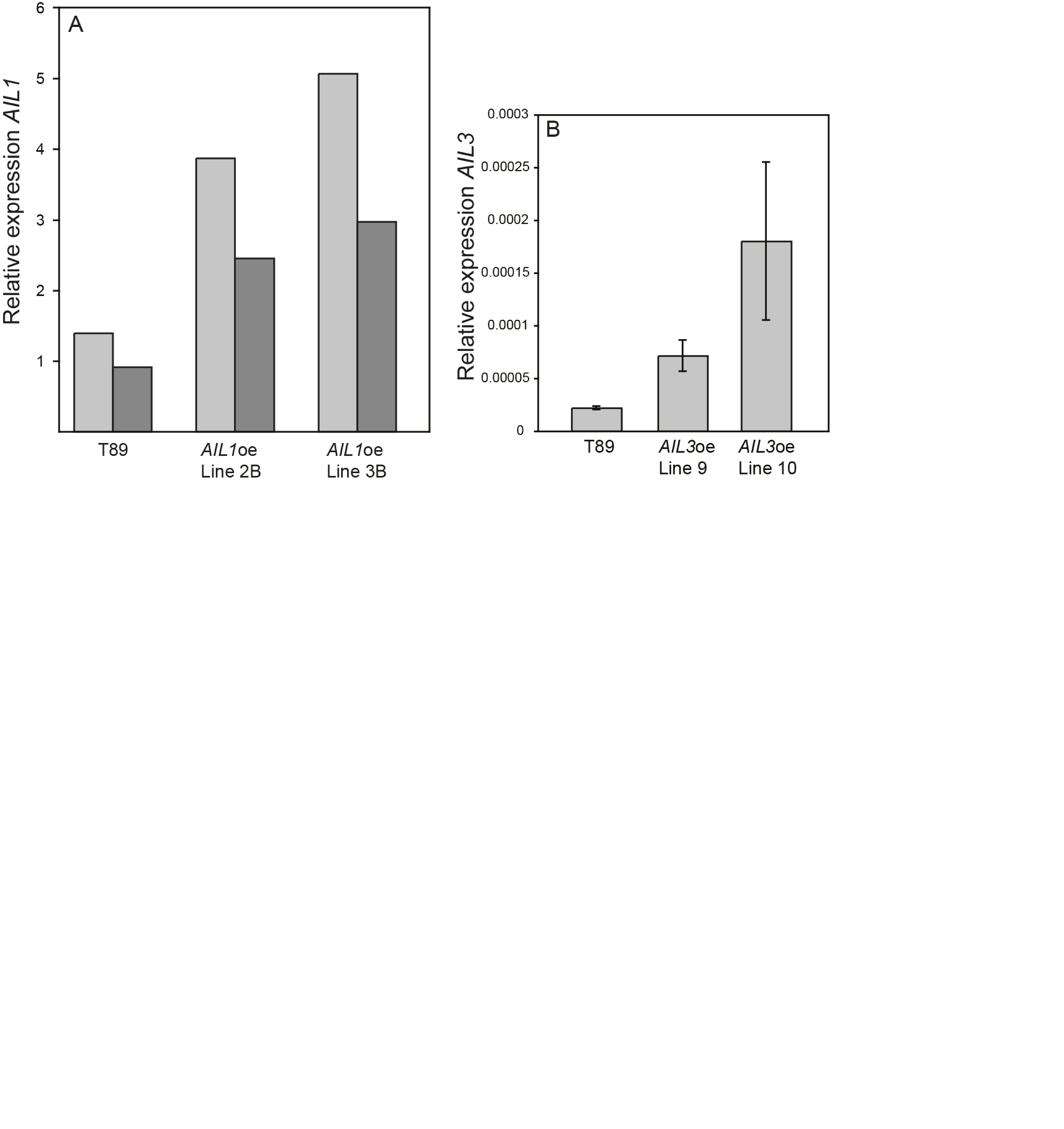

Supplement: Figure S3 — Expression of AIL1 and AIL3 in transgenic hybrid aspen. Expression in the apex of AIL1 in the wild type hybrid aspen (T89) and two transgenic lines (AIL1oe line 2B and 3B) expressing AIL1 cDNA under the control of 35S promoter. Y-axis shows the transcript levels of AIL1 relative to that of the reference gene (18S rRNA). Data from 2 independent experiments is shown. (B) Expression of AIL3 in wild type hybrid aspen (T89) and two transgenic lines (AIL3oe line 9 and line 10) expressing the AIL3 cDNA under the control of 35S promoter. Y-axis shows the ratio of AIL3 expression relative to that of the reference gene (18S rRNA). The expression values are average of 3 biological replicates and error bars represent the standard deviation for the three biological replicates. (TIF) [file pgen.1002361.s003.tif]

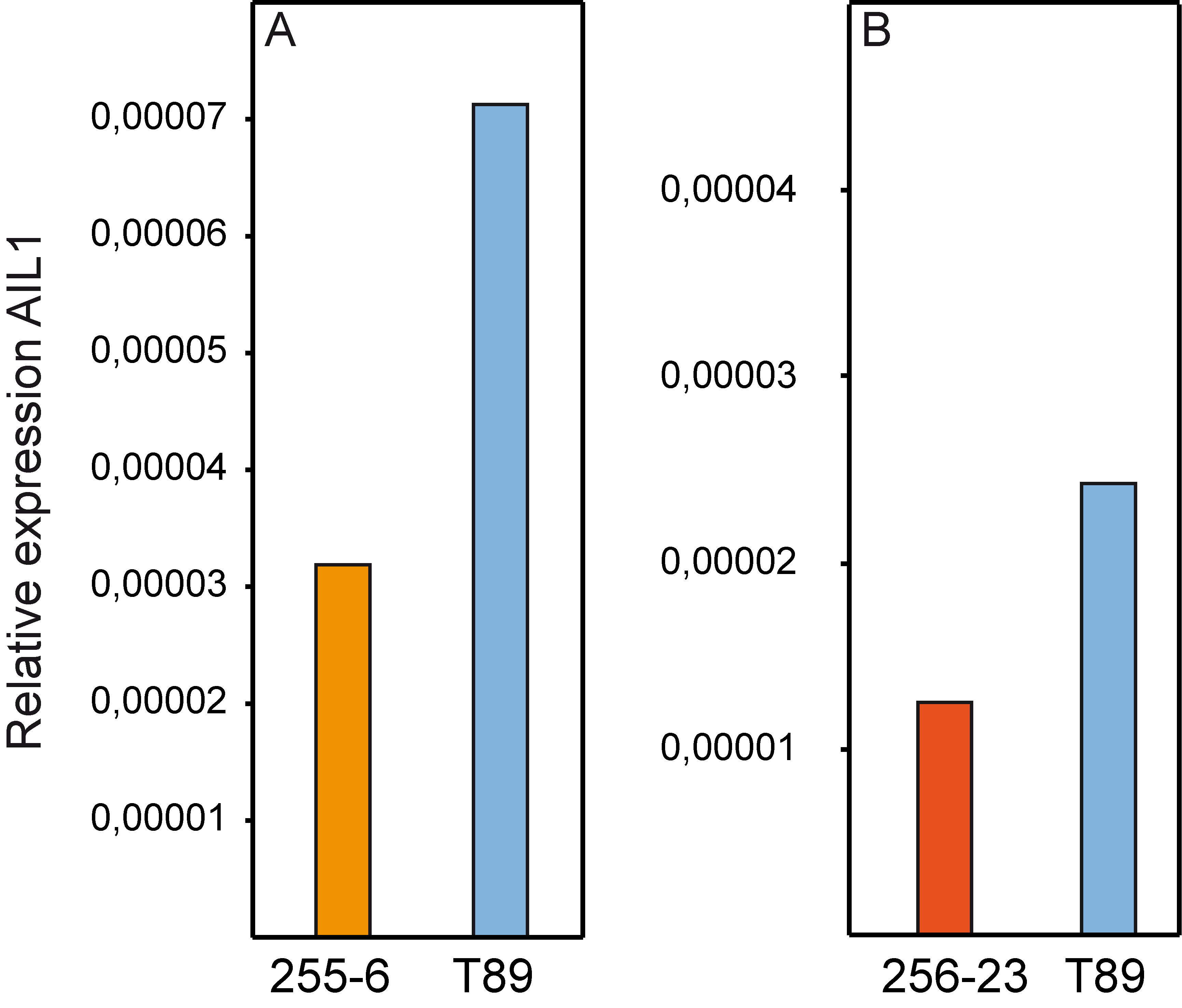

Supplement: Figure S4 — Expression of AIL1 in amiRNA expressing lines. A). Expression of AIL1 in apices of tissue culture grown wild type (T89) and amiRNA expressing hybrid aspen (255-6). A). Expression of AIL1 in apices of tissue culture grown wild type (T89) and amiRNA expressing hybrid aspen (256-23). Expression values are median of three technical replicates. (TIF) [file pgen.1002361.s004.tif]

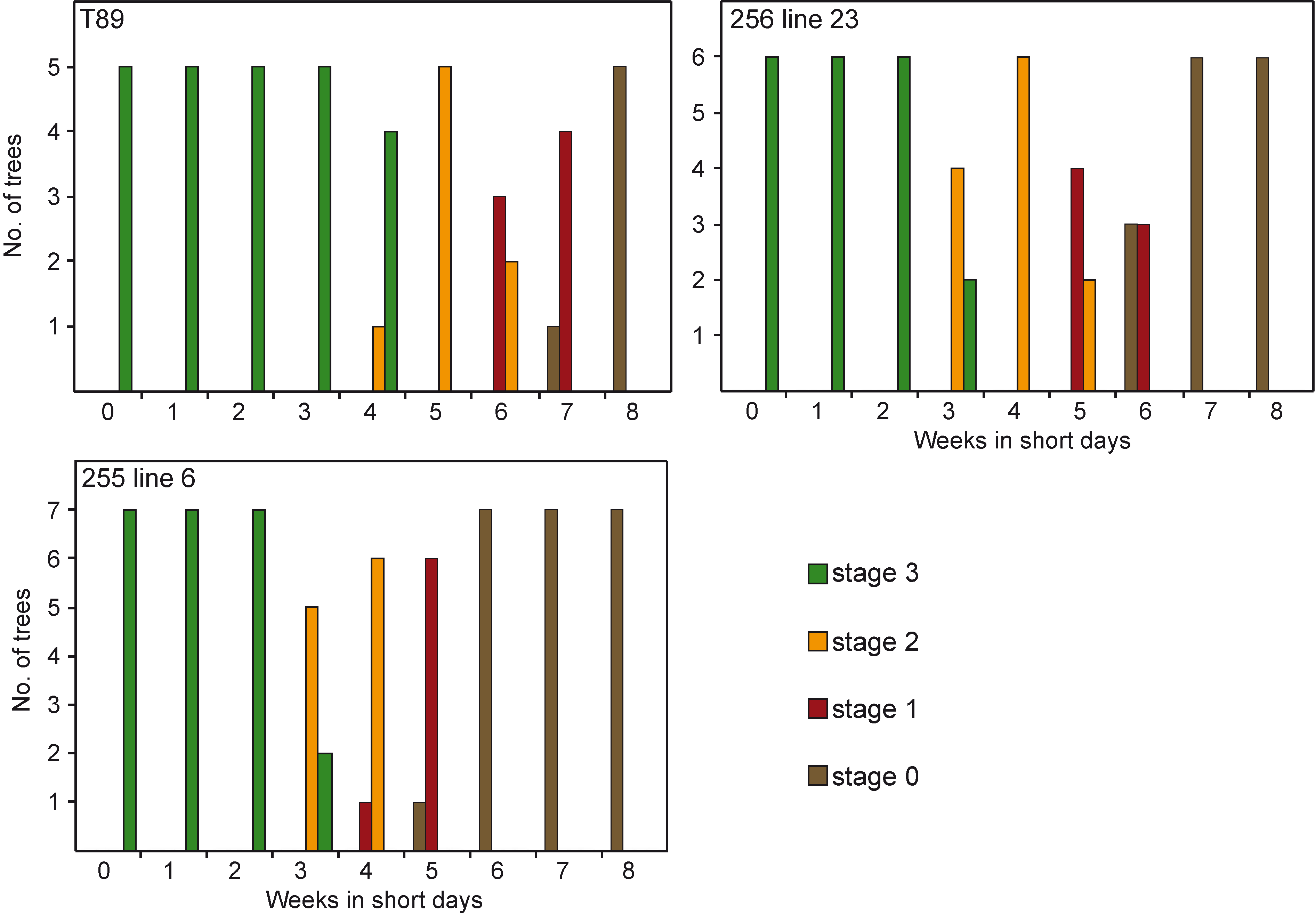

Supplement: Figure S5 — Analysis of bud set in amiRNA expressing lines. Analysis of bud set phenotype in wild type hybrid aspen (T89), and amiRNA lines 255-6 and 256-23. The transition from active growth to completion of bud set was divided into 4 stages where 0 is a completely developed bud and 3 correspond to an actively growing apex. Number of plants used for each genotype are: T89 n = 5, 255-6 n = 7 and 256-23 n = 6. X-axis denotes weeks in short days and Y-axis denotes number of plants at a particular stage of bud set transition. Colors specifying the stage of bud set are denoted. (Day length = 14h) (TIF) [file pgen.1002361.s005.tif]
