# Supplementary material for: Short Day–Mediated Cessation of Growth Requires the Downregulation of AINTEGUMENTALIKE1 Transcription Factor in Hybrid Aspen
Source: PLoS Genet. 2011 Nov 3;7(11):e1002361. doi: 10.1371/journal.pgen.1002361 (PMC3207903; doi:10.1371/journal.pgen.1002361)
Supplement: Table S1 — Real-time PCR primer sequences. *Please note that cross-reactivity could occur between primer pairs used for the detection of AIL1 and AIL2 gene expression. (DOCX) [file pgen.1002361.s006.docx]

| Target gene | Forward primer | Reverse primer |
| --- | --- | --- |
| AIL1* | TCTGTCTGTTATGCCCCTCA | CCACCTAGGAAGTCCTCCAGT |
| AIL2* | AAAATGGAGGCTGCTTCTGA | ACTTGTTGGAACGGCAGCA |
| AIL3 | GATGGGTCGCCTTGTATGAT | GTGATGGGTCCCCATAGTTG |
| AIL4 | CAGCAACTGGATCACAAAGC | AACTCGTGCTGGTGAACTGA |
| CYCD3:2 | GGATTTTCAGGTGGAGGACA | TGGATTCATCTTCCATTTAAGAGTAG |
| CYCD6:1 | AAGGGTTTCTTGCAACTTCG | CATGGCTTTGGTTGAGGAAT |
| FT2 | GAGGTTGTGTGCTACGAGAGC | CACTGTTTGCCTGCCTAGTTG |
| UBQ | GTTGATTTTTGCTGGGAAGCG | GATCTTGGCCTTCACGTTGT |
| 18S | TCAACTTTCGATGGTAGG | CCGTGTCAGGATTGGGTAATTT |
| TIP41-like | GCTGCACTTGCATCAAAAGA | GCAACTTGGCATGACTCTCA |
