# Supplementary material for: Short Day–Mediated Cessation of Growth Requires the Downregulation of AINTEGUMENTALIKE1 Transcription Factor in Hybrid Aspen
Source: PLoS Genet. 2011 Nov 3;7(11):e1002361. doi: 10.1371/journal.pgen.1002361 (PMC3207903; doi:10.1371/journal.pgen.1002361)
Supplement: Table S2 — Primers for CYCD3:2 promoter fragments. (DOCX) [file pgen.1002361.s007.docx]

| Fragment | Forward primer | Reverse primer |
| --- | --- | --- |
| pCYCD3:2 F1 | tcgaaacccacaaataaaac | TCGATGTCATCTTCTTCCA |
| pCYCD3:2 F2 | AATTTAACCCAATTCACAGC | GCCATCTCTCTCTCCTCTTT |
