# Supplementary material for: Short Day–Mediated Cessation of Growth Requires the Downregulation of AINTEGUMENTALIKE1 Transcription Factor in Hybrid Aspen
Source: PLoS Genet. 2011 Nov 3;7(11):e1002361. doi: 10.1371/journal.pgen.1002361 (PMC3207903; doi:10.1371/journal.pgen.1002361)
Supplement: Table S3 — Primers for amiRNA construction. (DOCX) [file pgen.1002361.s008.docx]

| Primer | Primer sequence |
| --- | --- |
| 255 I | gaTGTGTCTTGTAACACCTCTGTtctctcttttgtattcc |
| 255 II | gaACAGAGGTGTTACAAGACACAtcaaagagaatcaatga |
| 255 III | gaACCGAGGTGTTACTAGACACTtcacaggtcgtgatatg |
| 255 IV | gaAGTGTCTAGTAACACCTCGGTtctacatatatattcct |
| 256 I | gaTTGCAGGCCTCAACTGGGCTGtctctcttttgtattcc |
| 256 II | gaCAGCCCAGTTGAGGCCTGCAAtcaaagagaatcaatga |
| 256 III | gaCAACCCAGTTGAGCCCTGCATtcacaggtcgtgatatg |
| 256 IV | gaATGCAGGGCTCAACTGGGTTGtctacatatatattcct |
